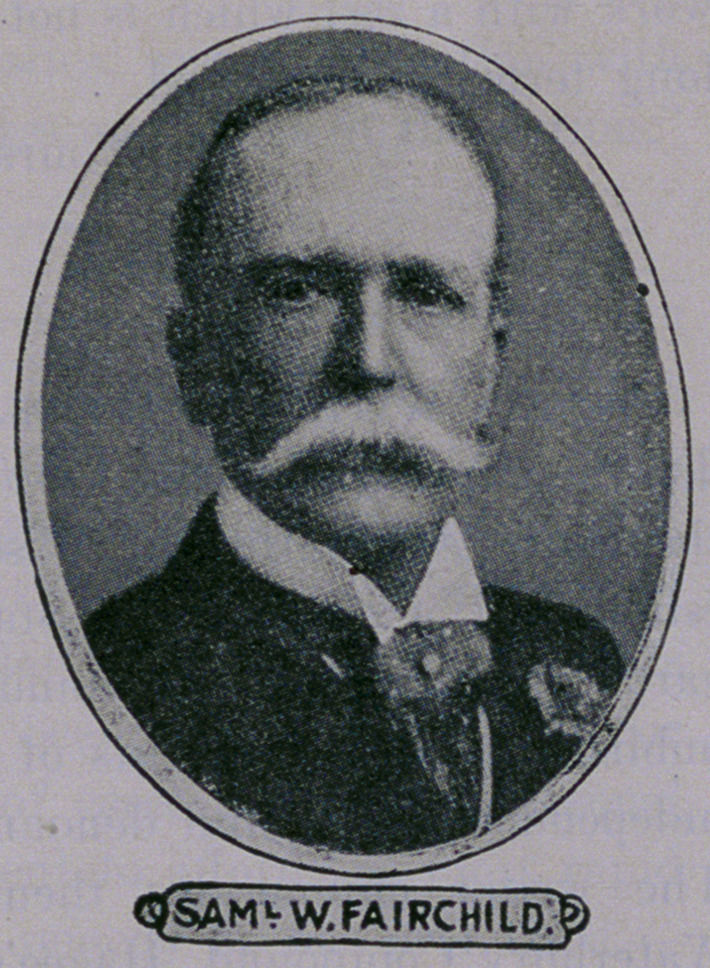# Editorial Notes, News and Miscellany

**Published:** 1912-09

**Authors:** 


### Editorial Notes, News and Miscellany.

MORPHINE, COCAINE, ABSINTHE AND WHISKY, in combination, can undo in an hour the civilizing influences of fifteen centuries.—*Sajous.*

WANTED.—A lady canvasser in every town to take subscriptions for the "Red Back," on liberal commissions. To canvass homes; women.

WANTED.—To correspond with some person going to Battle Creek. I will make it worth while. Editor "Red Back," Austin, Texas.

MR. RATTUS MUST GO; *delenda est.*—A war of extermination has been declared against him throughout the world. We surely do "hasten slowly."

AN INTERNATIONAL CONGRESS ON EUGENICS was held in London in July last, with an attendance of over four hundred delegates, representing twelve countries.

THE MEDICAL DEPARTMENT of the University of Texas has created a chair of Preventive Medicine, and Dr. J. P. Simonds, late of the Indiana State Board of Health, has been appointed to it.

PREPAYING SUBSCRIPTION to the "Red Back" to January, 1917—thirty-one consecutive years—Dr. S. Eagan, of Dallas, writes: "Your excellent JOURNAL is as valued and as valuable to me as ever,—a most welcome visitant, always."

DR. G. B. TAYLOR, eye, ear, nose and throat specialist, Cameron, Texas, spent four weeks in Chicago recently, "taking work" in his specialty. The doctor was accompanied by his wife and son, and they, I guess, took in the big show at the Colliseum.

DEATH OF DR. J. J. TAYLOR, OF PHILADELPHIA.—This distinguished physician and writer died August 2d (ult.), at Orange, N. J., at the early age of 58. He was the owner, editor and founder of the *Medical Council,* Philadelphia, one of the most readable journals published—almost unique in its style and character. He will be greatly missed from the journalistic field.

---

DR. JAMES GREENWOOD, JR., will, on September 10th inst., throw open the doors of his elegant and fully equipped sanatorium for the treatment of nervous diseases, drug addiction, etc., at Oak Heights, Houston, Texas. Dr. Greenwood is well qualified for this work, he having had a long training as assistant physician in the State insane asylums. See his announcement opposite first reading, this issue.

---

AN IMPORTANT AMENDMENT to the Pure Food and Drugs Law has just passed (August 20th) Congress. It includes, under the term "Misbranding," "any false statement, design or device regarding the curative or therapeutic effects of the contents of any package." It looks like that would knock out the sure cure patent medicines. Now, if Congress will make it unlawful for any newspaper to carry such advertisements it would be getting pretty close to one of the greatest evils of the day.

---

HOOKWORM PANDEMIC.—Dr. Boerner, the State Commissioner in charge of the hookworm work, says that 30 per cent of the people in Angelina county (East Texas, piney woods country), and 15 per cent of those of Trinity county are infested with hookworm. He has opened there numerous free dispensaries for the treatment of all who will apply. The treatment is simple and safe. A dose of Epsom salts in the morning, all day fasting, and 30 to 60 grains of thymol (in capsules) at bed time, a Seidlitz next morning, and "breakfast at ten," say the authorities.

---

THE GOAT'S "INNING."—The mosquito, the fly, the flea, the tick, the chinch, the rat, the dog, the squirrel, the skunk, the cat have all been found to be the host of disease-producing "germs," and now comes the goat to be added to the list. In Edwards county and Val Verde county, Texas, an epidemic prevails of what the authorities there call "Malta Fever," and they attribute it to a "germ" harbored by the goat. Those counties are large sheep and goat-raising regions, and it has been observed that the herders are those who suffer most. The State Board of Health has been appealed to to "investigate," and will do so at once. The goats were imported from Malta and the fever, which is infectious, is communicated through the milk.

MR. SAMUEL W. FAIRCHILD, of Messrs. Fairchild Bros. and Foster, of New York and London, whose generosity in connection with pharmaceutical education has been of such great assistance to students both in this country and America, and to whose liberality are due the advanced lectures on the essential oils now running at "The Square," has been paying his usual spring visit to London. Mr. Fairchild, with Mrs. and Miss Fairchild, has been staying at the Carlton Hotel, and left Southampton by the German Lloyd steamship "Kronprinzessin Cecilie" on his return voyage on Wednesday last.—*The British and Colonial Druggist,* May 31, 1912.

---

LETTER TO THE EDITOR.

JOHNSON CITY, July 4, 1912.

*Dr. F. E. Daniel.*

DEAR DOCTOR: The terrific rush of the present generation of youth to obtain an education in the shortest possible time is provocative of many ills, as the general practitioner is well aware, and in no sense prevalent instances is thus seen than in those of the little tots of from 6 to 12 years of age during the latter part of a six, seven, eight or nine months' term of school.

Brain-fag, eye-strain, headache and even physical deformity and worse from continued malposition of body, stunted growth and many ills, not thought much of by parents or teachers, but no less important are the results of a session which is prolonged beyond the mental and physical endurance of the younger students of our schools.

A division of sessions is the only remedy, in a seventh months' session three and one-half months for a session, a month's interval and then three and one-half months' teaching, and so on, in the sessions which are longer than six months. This division will still allow of our teachers attending the summer normals, and will be of immense benefit to both teachers and pupils, giving each that much needed rest in the middle of a school term—a rest which will enable each to begin, continue and finish his work with a zest which is not felt after perhaps two-thirds of such long terms have passed.

Yours fraternally,

GEO. HARWOOD, M. D.

---

CAUGHT WITH THE GOODS.—The Octopus (August 17th), under the caption *Noblesse Oblige*, editorially, bewails the fact that the State Association of Wisconsin, having fallen into line and established a "tentacle" (State medical journal), so far disregards the ukase of the immaculate Council of Pharmacy as to publish the advertisements of proprietary medicine carried by the independent press, and denounced and stigmatized as "nostrums." The writer enumerates them, as follows: Kutnon's Powders, Waterbury Compound, Hagee's Cordial, Ergoapiol, Champho-Phenique, Bovinine, Dioviburnum, Tongaline, Neurosine, and adds: "That the official organ of the State medical society of Wisconsin should give publicity to secret nostrums and encourage their use among its members, and that the State medical society itself should receive an income from such a source is not only a discredit to the organized profession of that State, but comes as a shock to those—they are many—who expect better things from Wisconsin."

The Octopus will never be able to force the seventy-odd thousand physicians outside of the ring to discontinue the use of the eight last named useful true and tried remedies. They are here to stay.

## Figures and Tables

**Figure f1:**